# Modulation of Light-Enhancement to Symbiotic Algae by Light-Scattering in Corals and Evolutionary Trends in Bleaching

**DOI:** 10.1371/journal.pone.0061492

**Published:** 2013-04-22

**Authors:** Luisa A. Marcelino, Mark W. Westneat, Valentina Stoyneva, Jillian Henss, Jeremy D. Rogers, Andrew Radosevich, Vladimir Turzhitsky, Margaret Siple, Andrew Fang, Timothy D. Swain, Jennifer Fung, Vadim Backman

**Affiliations:** 1 Department of Civil and Environmental Engineering, Northwestern University, Evanston, Illinois, United States of America; 2 Department of Zoology, Field Museum of Natural History, Chicago, Illinois, United States of America; 3 Department of Biomedical Engineering, Northwestern University, Evanston, Illinois, United States of America; Institute of Marine Research, Norway

## Abstract

Calcium carbonate skeletons of scleractinian corals amplify light availability to their algal symbionts by diffuse scattering, optimizing photosynthetic energy acquisition. However, the mechanism of scattering and its role in coral evolution and dissolution of algal symbioses during “bleaching” events are largely unknown. Here we show that differences in skeletal fractal architecture at nano/micro-lengthscales within 96 coral taxa result in an 8-fold variation in light-scattering and considerably alter the algal light environment. We identified a continuum of properties that fall between two extremes: (1) corals with low skeletal fractality that are efficient at transporting and redistributing light throughout the colony with low scatter but are at higher risk of bleaching and (2) corals with high skeletal fractality that are inefficient at transporting and redistributing light with high scatter and are at lower risk of bleaching. While levels of excess light derived from the coral skeleton is similar in both groups, the low-scatter corals have a higher rate of light-amplification increase when symbiont concentration is reduced during bleaching, thus creating a positive feedback-loop between symbiont concentration and light-amplification that exposes the remaining symbionts to increasingly higher light intensities. By placing our findings in an evolutionary framework, in conjunction with a novel empirical index of coral bleaching susceptibility, we find significant correlations between bleaching susceptibility and light-scattering despite rich homoplasy in both characters; suggesting that the cost of enhancing light-amplification to the algae is revealed in decreased resilience of the partnership to stress.

## Introduction

Reef-building scleractinian corals depend on algal symbionts for daily energy requirements [1] and have evolved strategies to harvest light in heterogeneous environments, including modulation of the density of algae and cross-absorption of the algal photosynthetic pigments [2], [3], adoption of efficient colony morphologies [4], [5], and production of fluorescent pigments to dissipate or enhance light availability [6], [7]. Corals also construct highly reflective calcium carbonate skeletons that diffusely backscatter unabsorbed light back toward the algae, amplifying light available to the algal photosynthetic complex by 3–20 times relative to incident light levels [8]–[10]. Although beneficial under typical irradiances [8], thermal stress may cause excess light to lower the temperature and time thresholds for bleaching [9] through a mechanism similar to that observed for corals exposed to high irradiances [11], [12]. The potential trade-offs between benefits (increased photosynthetic activity) and costs (potential bleaching) of light-amplification make this physiological system essential to understanding coral-algal physiology, distribution, evolution, and conservation. Here we used a novel optical spectroscopic technique, low-coherence enhanced backscattering (LEBS), originally developed for early cancer detection [13], to determine optical and structural properties of coral skeletons. We explore how corals control light-amplification from optical, structural, and evolutionary perspectives and demonstrate its association with taxon-specific susceptibility to bleaching and death.

## Results

### Which light-scattering properties of coral skeletons modulate light-amplification to symbiotic algae?

**Figure 1:**
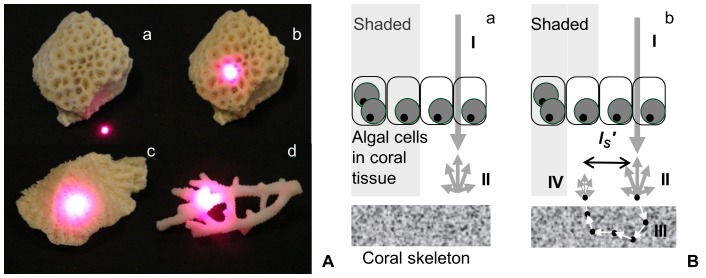
Light transport in coral skeletons. **A** – Visual demonstration of differences in light transport shown for three taxa as described in [10] by focusing a laser on (a) highly-absorbing black surface and on skeletons of (b) *Leptastrea transversa*, (c) *Leptoria phrygia*, and (d) *Seriatopora caliendrum*. Microscopic light-scattering properties of skeletons were measured using LEBS with a white light source. **B** – Schematic representation of the redistribution of light between sun-exposed versus shaded areas. Differences in light transport are shown for corals with (a) very high 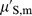 skeleton and a (b) low 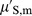 skeleton. Skeletons capable of longer light transport (i.e. longer 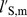 or low 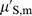) are able to illuminate otherwise shaded areas in the colony and this increased redistribution between sun-exposed versus shaded areas of a colony may further amplify the light available to the algae: (I) downwelling light, (II) diffuse reflectance, (III) photon path (arrows) and sub-micron scatters (black dots), (IV) diffuse reflectance illuminating a shaded algal cell in the coral tissue: the skeleton serves as a secondary light source [9].

Amplification depends on the diffuse reflectance of light from coral skeletons [9], [14] where scattering is a key mechanism behind reflectance (Fig. 1A). In coral skeletons, as in any turbid medium, scattering is due to light interaction with microstructures (∼30–1,000 nm, hereafter ‘microscopic-scattering’ [15]) ranging from nanometers (e.g. 50–200 nm CaCO_3_ nanograins) to microns (e.g. 1–5 µm ‘fiber bundles’ [16], [17]). Amplification is further modulated by light-reflection from larger length-scale structures; from micron-size septa to millimeter-size corallites [9], [10], [14]. Microscopic-scattering is not affected by voids such as those between septa, although these spaces do affect ‘bulk-scattering’ properties. In order to isolate the fundamental microscopic-scattering properties irrespective of taxon-specific morphological differences (e.g. corallite diameter, complexity, and density), we used LEBS to focus on shorter photon path lengths at the level of microstructures (∼100 µm) thus reducing effects of bulk-scattering. LEBS measures a key microscopic-scattering property of skeletons, reduced scattering coefficient (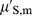), which is the inverse of the distance a photon travels until it becomes randomized in direction; i.e. transport mean free path length, 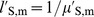 [18].

We measured 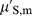 of 150 coral skeletons representing 96 Atlantic and Indo-Pacific taxa with various colony morphologies (Table S1). Mean 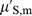 per skeleton was obtained by measuring 20 sites on each skeleton (LEBS spot diameter was ∼1.5 mm); thus averaging over major morphological structures such as corallites, septa, columella, and coenosteum; and mean 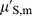 per taxa was obtained by measuring 1–8 independent colonies per taxon (Table S1). The mean 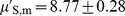 mm^−1^ (mean ±SE; Fig. S1) was greater than previously measured bulk values (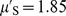 mm^−1^ [14]) most likely because it is determined by highly scattering solid structures without the effect of voids. The magnitudes of 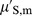 and 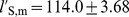 µm have important physiological consequences. For example, as light hits a septum in a corallite scattering within the septum leads to light-amplification for the proximate symbionts, especially in the case of corals with high-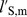. Because the extent of light transport is a multiple of 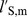 (∼millimeters), light will diffuse into neighboring septa and redistribute throughout the colony. This enables millimeter-size structures to increase amplification as much as 10–20-fold by trapping light within coral tissue due to multiple passes [8], [9]. This mechanism of redistribution also delivers light to shaded parts of the coral colony (Fig. 1B). Thus, at optimal values of 114 µm, 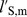 is sufficiently long to redistribute light but short enough to minimize loss of light from the structure to ensure the high skeletal reflectivity required for amplification [2], [8], [9].

### How is light-scattering related to coral bleaching susceptibility?

**Figure 2:**
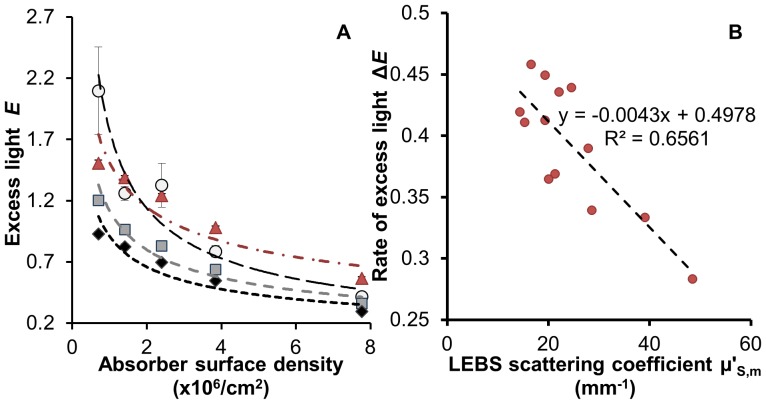
Excess light dynamics. **A** – Relationship between excess light (*E*) and concentration of absorbing particles (*ρ*). Data collected using ‘flat coral models’: Bottom layer: ∼1 mm skeleton slices (*Pocillopora damicornis* - open circles; *Seriatopora hystrix* - squares, *Porites lobata* - diamonds) on top of a highly scattering standard or the standard alone - triangles. Top layer: set of five 1 mm polymer layers containing progressively lower concentrations of fluorescent 6 µm microspheres (*ρ*) mimicking light absorbing symbionts densities in healthy tissue (100% cover = 7.8×10^6^ microspheres/cm^2^) and in corals undergoing bleaching response up to 93% bleached (0.7×10^6^ microspheres/cm^2^). **B** – 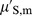 association with the rate of excess light increase (Δ_E_) for 13 skeletons of 10 coral species. Δ_E_ was calculated for each ‘flat coral’ construct from data as in Fig. 2A.

We measured the relationship between scattering and amplification using a ‘flat coral’ model to simulate a bleaching response. These data suggest that excess light *E*, defined as the difference between light intensities experienced by symbionts (with and without a skeleton) normalized by the intensity without the skeleton (see Text S1 section 1.6.), increases when symbiont concentration (*ρ*) decreases (average r^2^ = 0.95, Fig. 2A). This supports the hypothesis that as symbionts are lost in response to thermal stress, light-amplification can further magnify the stress on the remaining algae leading to a positive feedback-loop that accelerates bleaching [9]. Importantly, the *rate* of excess light increase, 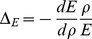, was inversely related to 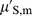
(Fig. 2B, r^2^ = 0.66). Although 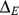 depends also on the absorption coefficient, 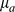, for a typical coral skeleton 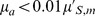 and is too small to affect light transport at length scales ∼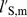 (Fig. S2). Consequently, when low-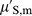 (i.e. high 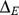) corals bleach, the excess light *E* rises more rapidly compared to high-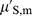 corals, thus exposing the remaining symbionts to even greater light intensities and leading to an earlier or more pronounced bleaching response. We therefore hypothesized that 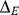 should correlate with coral bleaching response. This does not necessarily imply a correlation between steady-state *E* and bleaching, as corals may acclimate to a higher light environment; instead, it is a positive feedback-loop measured by 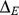 that is expected to adversely affect coral response to stress (see Text S1 section 1.6.).

**Figure 3:**
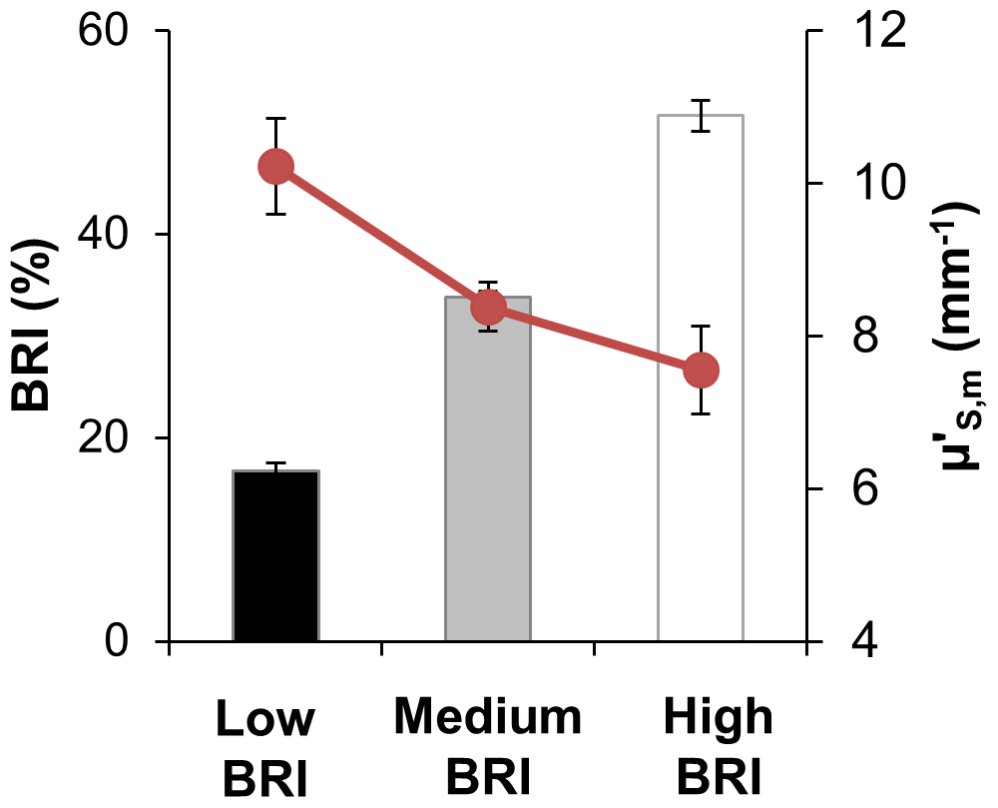
Relationship between 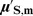 and bleaching response index (BRI). Data organized into low (31 taxa, BRI = 18.42±0.82%, mean ±SE), medium (48 taxa, BRI = 36.35±0.53%) and high (17 taxa, BRI = 57.27±1.5%) BRI clusters; ANOVA, p<0.002.

We tested whether coral species with low 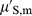 skeletons show an increased susceptibility to bleaching. We designed an empirical bleaching response index (BRI) from a meta-analysis of 1,412 independent taxon-specific records of coral bleaching severity and bleaching-related mortality throughout the tropics which were compiled from literature and digital datasets collected in 1982–2006 (Tables S2, S3). For each of the 96 taxa in this study, BRI was defined as the average percent of taxon-specific coral cover that was affected by bleaching (i.e. bleached or dead; Table S1). We grouped taxa into three clusters based on BRI values (low, medium, and high) via k-means clustering (Table S1). There is an inverse relationship between BRI and 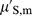 (Fig. 3) supporting our hypothesis (ANOVA, p<0.002; linear regression p<0.01, which remained significant after accounting for the potential confounding effects of colony morphology; see Text S1 section 1.5.).

**Figure 4:**
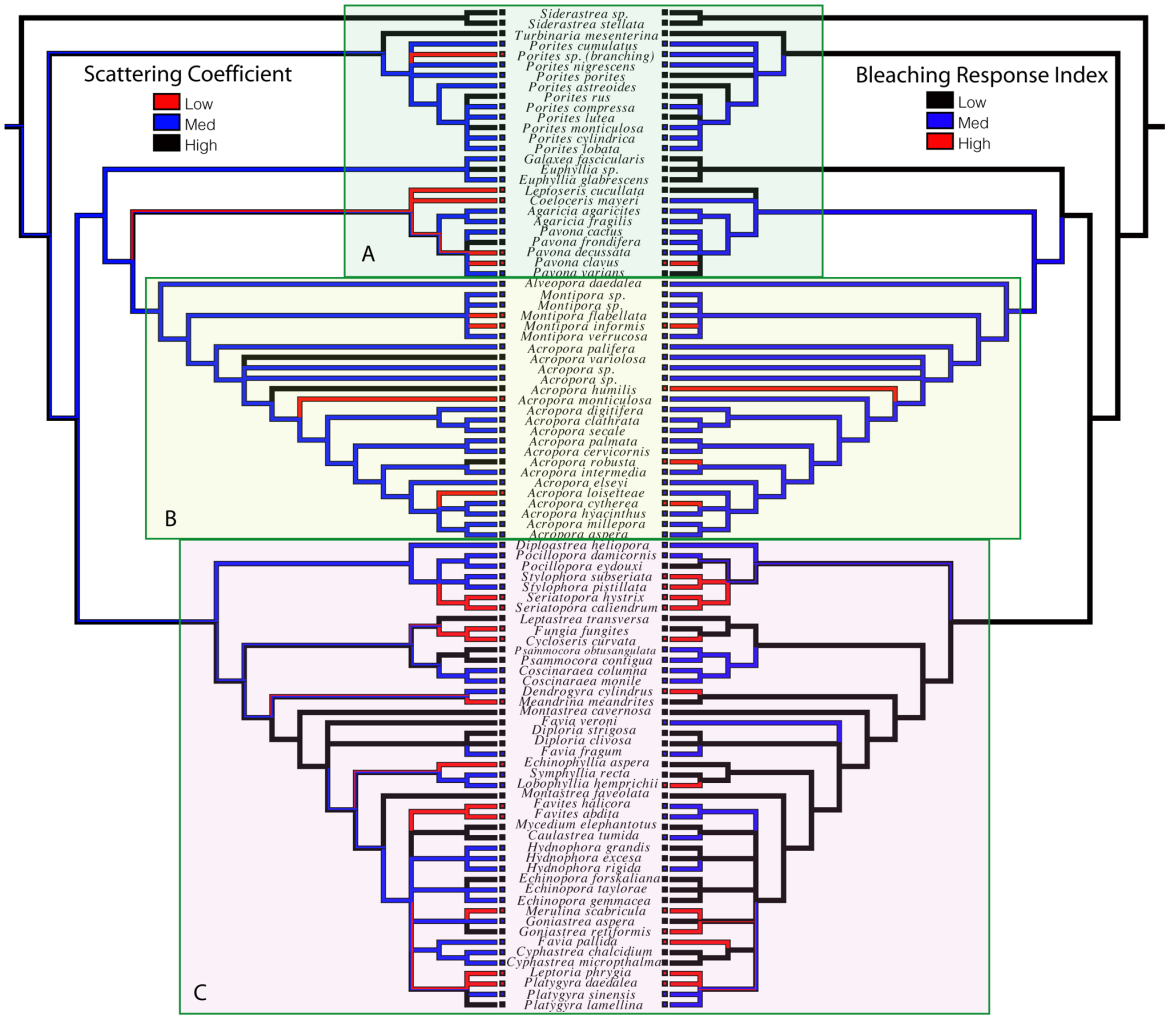
Evolutionary correlation between scattering coefficient and coral bleaching. A composite phylogeny shown in mirror image, with character states for 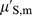 (left) and BRI (right) mapped to illustrate their significant correlation (p<0.05) throughout the evolutionary history of corals. High bleaching susceptibility appears to be less common toward the base of the coral tree (box A) and higher in the *Montipora-Acropora* clade (box B) and the “Robusta” coral clade (box C).

We then investigated the pattern of evolutionary change in bleaching susceptibility and light-scattering by mapping these traits onto a composite phylogeny of corals (Fig. 4) [19]–[23]. Phylogenetic independent contrasts tests of correlation between bleaching (BRI) and scattering (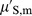) showed a significant negative correlation (r = −0.20, p<0.05), supporting the hypothesis that decreasing 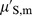 is associated with increasing BRI regardless of evolutionary relatedness. Bleaching and scattering show a strong pattern of independent origins and/or reversals many times during the evolutionary history of corals. Clades that diverged early in coral history (Fig. 4, box A) have low to medium BRI and medium to high 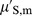 values. The *Acropora* clade (Fig. 4, box B) has medium to high BRI values, with several examples of high susceptibility. The frequency of evolutionary change in bleaching risk and light scattering appears to be highest in the “robusta” clade (Fig. 4, box C), with members of 10 different genera showing high bleaching susceptibility. Most coral clades have species that span the range of bleaching susceptibility from low to high, with the emergent pattern being a mosaic of character distributions, with up to 12 evolutionarily independent origins of high bleaching susceptibility in our species sample.

### How do corals control light-transport?

**Figure 5:**
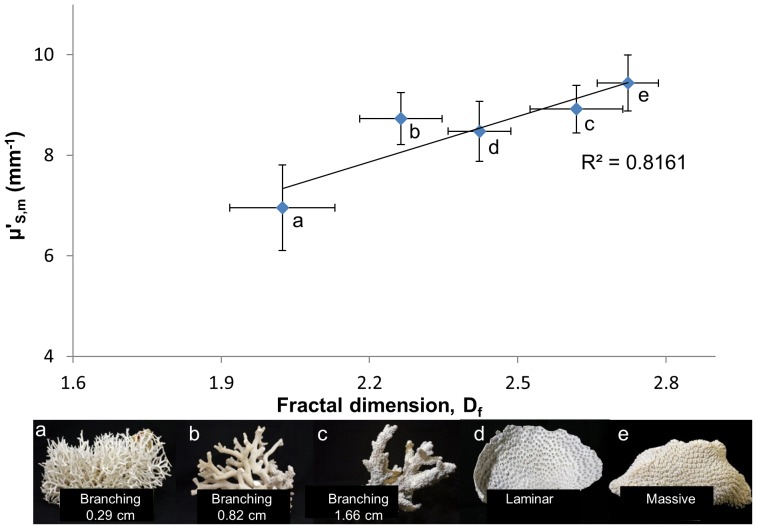
Relationship between growth-form averaged-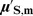 and fractal dimension (D_f_). Example colonies of various growth forms: (a) thin-branching: *Seriatopora hystrix*, (b) medium-branching: *Stylophora subseriata*, and (c) thick-branching *Acropora variolosa* (average diameter of branches shown in figure), (d) laminar/foliaceous: *Echinopora lamellosa* and (e) massive: *Galaxea* sp.

**Figure 6:**
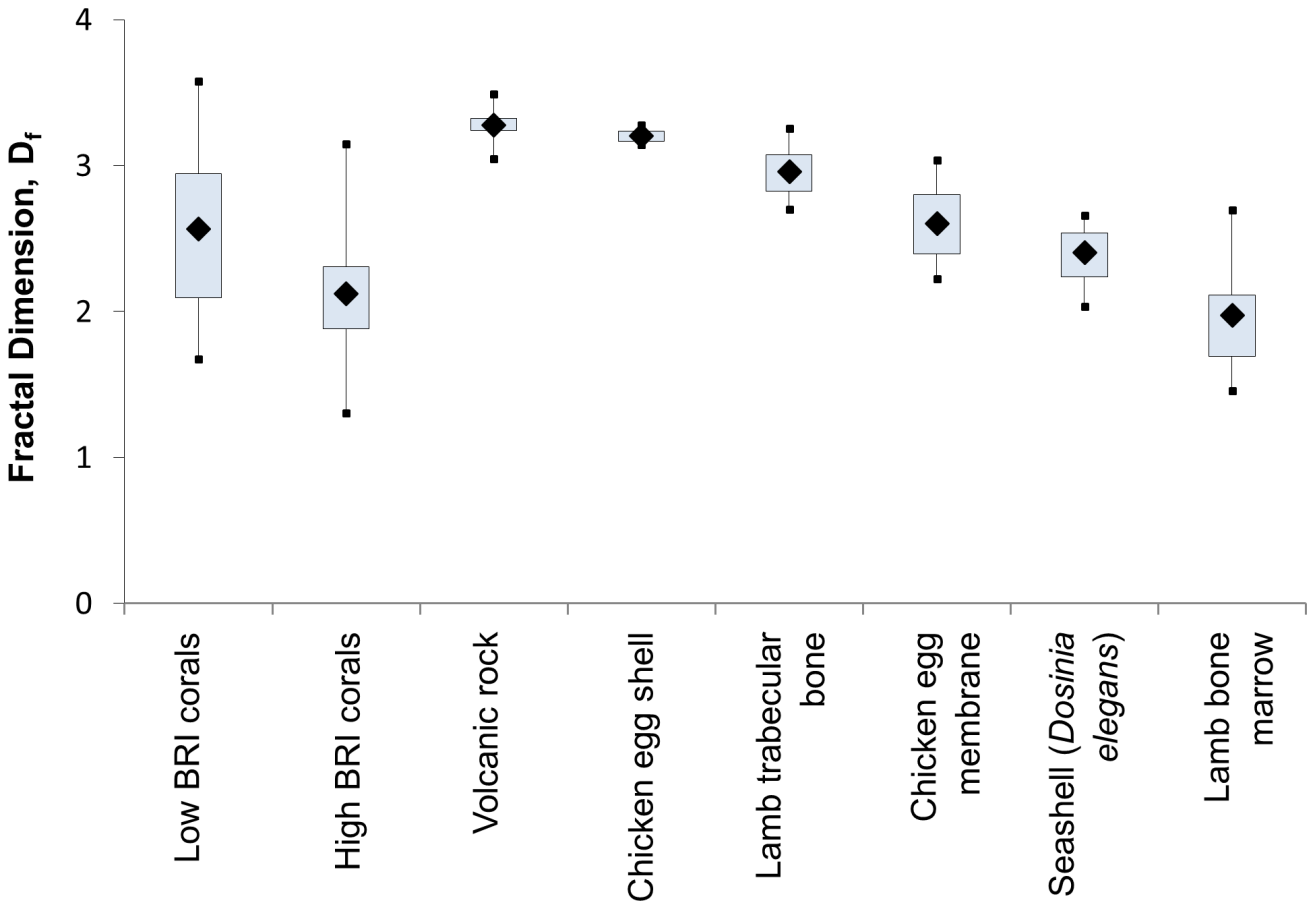
Fractal dimension of different biogenic (biomineralized) and non-biogenic materials as measured by LEBS.

The vast majority (90%) of the 150 skeletons examined had micro-morphology (30–1,000 nm structures) consistent with a ‘mass-fractal’, i.e. a structure with a similar degree of compactness at various length-scales [24], [25] (average mass-fractal dimension D_f_ = 2.44±0.04). As predicted by Born approximation, the light-scattering cross-section of a particle increases with its size and a medium with higher D_f_ would have a shift of its structures toward larger length-scales thus leading to a higher 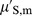 [15]. Our data confirm an increase of 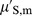 with D_f_ (linear regression, p<0.01; Fig. 5; see Text S1 section 1.5.) and are within the fractality range of other biomineralized structures (Fig. 6) [26]–[30]. Skeletal fractality may reflect coral physiology and skeletogenesis and also represent an optimal growth strategy by exhibiting strong morphological plasticity in response to variable light intensities and nutrient flow-rates [25]. D_f_ describes the complex dynamics of skeleton formation where linear extension and increased density occur by infilling of spaces [16], [17]; a lower D_f_ corresponds to a shorter average length-scale of skeletal nano-/microstructures due to a higher rate of linear extension as compared to the rate of infilling [16], [17], [31] typical of corals with higher growth rates. In our dataset, branching corals (n = 65) had a lower D_f_ than massive corals (n = 39; D_f_ = 2.29±0.44 *versus* 2.72±0.41, mean ±SE), which is concordant with their higher growth rates (58.93±36.8 *versus* 6.91±3.56 mm/year, mean ±stdev) [32]–[35].

## Discussion

This research indicates that corals with lower mass-fractal dimension D_f_ have lower reduced scattering coefficient 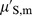 and higher light-amplification rate 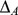. Under normal environmental conditions low-D_f_/low-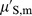 may be advantageous as these corals often exhibit faster growth rates. However, once bleaching is initiated, these properties further enhance stress on the remaining algae thus accelerating the bleaching response.

It will be important to understand whether the variability in light collection efficiency across coral taxa influences the establishment and maintenance of symbiosis with diverse *Symbiodinium* adapted to particular light regimes and capable of conferring differential degrees of fitness (e.g. growth, survival, and thermotolerance) to the holobiont (e.g. [36], [37]). Recent evidence unveils a complex interplay between *Symbiodinium* phylotypes (which may affect host physiology by modulating its transcriptome, growth, and response to thermal stress [37], [38]) and the host (which may modulate the heat/light tolerance of *Symbiodinium* through autofluorescence proteins or skeleton backscattering, e.g. [6], [7], [14], [39], [40]) and determine clade composition [38]. It is clear that both partners contribute with protective mechanisms to reduce the damaging effects of heat and light stress (e.g. [6], [11], [40]), but the relative contribution of each partner to the overall fitness of the holobiont remains largely unknown.

This research indicates that the coral host can influence the bleaching response and (potentially) overall growth by the amount and rate of light increase that is scattered by the skeleton back to the algae. Furthermore, the variability and apparent lability of light-scattering properties across the phylogeny of corals provides a promising system in which to test multiple independently derived examples of light handling strategies in the context of their risk of bleaching and their priority for conservation. The challenge now is to identify the host-symbiont interactions that contribute to the overall fitness of the holobiont to better predict the response of coral reefs to increased stress due to global climate change.

## Methods

### Coral skeletons

Coral skeletons were obtained from the Field Museum (Division of Invertebrates; n = 91) and the Smithsonian Institution (U.S. National Museum of Natural History, Invertebrate Zoology Department; n = 59) collections representing 89 Atlantic and Indo-Pacific species and 7 specimens identified at the genus-level (Table S1). Pieces of colonies (1–3 cm^2^) were sampled using a Foredom® flex-shaft system drill with 220-grit diamond wheel bit. Corals were classified as belonging to one of four growth form categories: Branching (n = 65), Laminar/Foliaceous (forming either plates or tiers and whorls; n = 45), Massive/Encrusting (boulder and encrusting forms; n = 38), and Solitary (n = 2). Branching corals were further classified as thin, medium, and thick forms based on the average size of branch diameter measured at the base, tip, and middle of 5 branches per colony. Medium-branching forms were classified as those with diameters within the average ± stdev (1.31±0.60 cm) of all branching corals, and thin and thick forms were defined as diameters <AVG−1 stdev (<0.71 cm) and >AVG+1 stdev (>1.91 cm), respectively. Corals were assigned to biogeographic realms [41] by sampling location. Specific reef location and depth data were not available.

### Low Coherence Enhanced Backscattering (LEBS) spectroscopy

The LEBS instrument has been described in detail in other publications [13], [18], [42] and it is further described in Text S1 (section 1.1). The microscopic reduced scattering coefficient 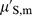 can be measured from the LEBS enhancement factor 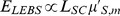 (see Text S1 section 1.1.). The micro-architecture organization was analyzed by characterizing the optical refractive index correlation function C(r) for length scales *r* from ∼30–1,000 nm. Because optical refractive index is a linear function of local mass density, C(r) is proportional to the autocorrelation function of spatial mass-density fluctuations (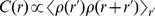, where ρ is the local density of the material and brackets 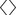 designate average over position r′ in the three-dimensional space, and r the distance between correlated structural elements [43]. The mass-density autocorrelation function C(r) is a measure of spatial mass distribution that quantifies correlation between densities separated by distance r, thus characterizing the size distribution of the structural elements. A functional form of C(r) can be quantified by LEBS via parameter 
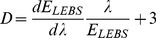
, with 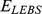 and *λ* (the wavelength of light) taken as the average over the wavelength range of interest. Here, the parameter *D* quantifies the shape of C(r): if D<3, *C*(*r*) is a mass fractal (in case when the upper limiting length scale *l_c_* of fractality is such that 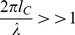, a condition that is typically satisfied in most biological tissues including coral skeletons) with mass-fractal dimension D_f_ = D; 3<D<4 corresponds to a stretched exponential correlation function, D = 4 – exponential and D>>4 – Gaussian correlation [13]. In case of a mass-fractal morphology, D_f_ is directly related to the average length-scale of skeletal nano-/micro-structures (referred to as ‘coherence length’ in [31]). In essence, a skeleton with a higher D_f_ would have on average the size distribution of its nano- and micro-structures shifted toward larger sizes.

A short Lsc (∼30–300 microns in our instrument) ensures that the LEBS signal is generated only by photons that propagate paths comparable to Lsc within a coral skeleton. At these length scales, skeletons consist of dense skeletal tissue (e.g. septa) separated by voids filled by coral tissue (e.g. spaces between septa within a corallite) [16], [17], [35]. The photon paths can then be tracked within these solid structures while ignoring the contribution of photons that leak out of these structures, which would otherwise make bulk-scattering properties dependent on the skeletal macro-architecture (e.g. corallite diameter and density, number of septa). Specifically, we focused on path lengths within ∼100 µm.

The mass-fractal dimension (D_f_) of several biomineralized structures; e.g. calcareous eggshell (chicken eggshell), calcium-phosphate (hydroxyapatite) bone and bone-marrow (trabecular bone and bone marrow of lamb), and a calcareous seashell (*Dosinia elegans*) as well as a non-biomineralized structure such as igneous rock; were measured using LEBS and compared with the average D_f_ of the low and high BRI corals (Fig. 6). See additional information about LEBS in Text S1 section 1.1.

### Measurement uncertainty

Measurement uncertainty was estimated for the reduced scattering coefficient (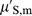), mass fractal dimension (D_f_). and Bleaching Response Index (BRI) as detailed in the Text S1 section 1.2.

### Measuring light amplification to the algae using a ‘flat-coral model’

A coral model system was developed consisting of a tissue-mimicking layer composed of fluorescent microspheres (Thermo Scientific, 36-2, absorption peak at 542 nm and emission at 612 nm) embedded in polymer resin (polydimethylsiloxane, PDMS, index of refraction close to tissue, n = 1.41, Slyguard 184, Dow Corning Corp.) placed on top of a 1–2 mm thick slice of coral skeleton. The fluorescent microspheres were mixed with the PDMS elastomer base in different concentrations, sonicated for several hours for even dispersion, followed by adding the curing agent and degassing in a vacuum oven for 10–15 min or until no more air-pockets were present [44], [45]. The air-free mixture was heat cured at 60°C for at least 4 hours. To produce uniform thickness and minimal surface roughness, the resin solution was cured between 2 glass plates with 1 mm spacers between the plates. After removing the glass plates the tissue-mimicking layer surface was approximately 2×5 cm.

For consistency with the range of algae sizes reported in the literature, microspheres with 6 µm diameter were selected. Coral bleaching was simulated by constructing five tissue-mimicking layers with progressively lower concentrations of microspheres (7.76, 3.84, 2.4, 1.41 and 0.7×10^6^ microspheres/cm^2^ corresponding to healthy coral, 52, 74, 86, and 93% bleached coral respectively).

Each layer was placed on top of coral skeleton samples from 10 different species of the Field Museum collection prepared by cutting 1–2 mm slices with a diamond saw, further thinned and polished by lapping to reduce surface roughness (total of 13 slices). A reflectance standard (hereafter ‘white standard’; Labsphere, SRS-99-010) was included in the experiment as an example of an extreme case of a highly scattering medium. The white standard has a very high reduced scattering coefficient *μ′_s_*∼50 mm^−1^ relative to that of the coral skeletons (average 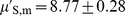 mm^−1^) with reflectance *R*∼1.

There are several limitations of our model in mimicking the optical properties of the coral tissue. Firstly, the absorption efficiency (absorption cross-section normalized by the geometrical cross-section) of the fluorescent microspheres (0.15) is smaller than that of zooxanthellae (0.98) [14]; thus, for the same density of absorbers, our tissue-models absorb less than coral tissue. Secondly, the fluorescent microspheres have comparable absorption and scattering cross-sections while the zooxanthellae have much smaller scattering cross-section leading to some light transport due to scattering in the top absorbing layer in our models compared to coral tissue. This effect, however, is relatively minor because 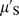 of our tissue models (ranging from 0.03–0.35 mm^−1^) is substantially lower than 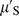 of the 13 skeleton layers studied (ranging from 14.5–39 mm^−1^). A mathematical model of light amplification is also described in detail in the Text S1 section 1.3.

### Measuring optical properties and light amplification using an integrating sphere technique

The optical properties of the tissue models were characterized using the integrating sphere (IS) technique similar to [2], [14] in combination with the Inverse Adding-Doubling (IAD) method [46]–[48] based on the diffusion approximation and radiative transport theory, and are described in detail in Text S1 section 1.3. and Fig. S3.

### Bleaching response index (BRI)

Using taxon-specific data from mass bleaching events throughout the tropics from 1982–2005 that were previously published in peer-review, gray literature, and electronic databases (Table S3) we compiled 1,412 unique records of bleaching severity and related mortality (Table S2). All collected data were converted to a bleaching response index (BRI) which was defined as the taxon-specific average percent coral cover affected during mass bleaching events, i.e. coral colonies found bleached and/or dead. Detailed information of the construction of BRI is provided in Text S1 section 1.4, with an example of the conversion from disparate indices in Table S4. Taxa were further assigned to a number of severity categories that were defined based on clustering analysis for BRI (Mathematica, k-clustering algorithm) and the detailed description is provided in Text S1 section 1.4.

### Phylogenetic Analysis

The phylogeny used in our analysis was assembled based on recently published molecular and morphological studies [19]–[23]. We built a composite phylogenetic tree of coral evolutionary relationships for the 96 taxa used in this study. The main structure of the tree follows that given in Fig. 1 of Fukami et al. [19] based on molecular sequence data from cytochrome oxidase I and cytochrome b mitochondrial genes. Additional information was also incorporated from trees based on molecular sequence data from the ß-tubulin gene and a portion of the nuclear ribosomal DNA (rDNA) containing the 3′- end of 18S, internal transcribed spacers, 5.8S, and the 5′- end of 28S [19], [23].

Taxa in our skeleton collection that were not found in the Fukami et al. [19] tree where placed in the tree according to the position of other taxa in the same genus. However, there were some exceptions to this general rule. There was no representative of the genus *Cycloseris* in the tree in [19]; therefore *Cycloseris curvata* was placed in our phylogeny according to its position in the super tree of Kerr [20]. The lack of resolution in the Faviidae family lead to difficulty positioning *Favia veroni*, as there was no molecular data for this species. Therefore, *Favia veroni* was placed as a basal member of the clade that contains all other *Favia spp.* Little information was found on the phylogenetic position of the genus *Coeloceris*, therefore *Coeloceris mayeri* was placed as a basal member of the Agariicidae clade based on its association with the family [49]. Additional resolution was also added to the *Acropora* clade and the *Porites* clade based on the morphological analysis of Wallace and the molecular analysis of Forsman et al. [21], [50].

In order to assess the robustness of the patterns of correlation between bleaching susceptibility and backscattering variables, we analyzed many phylogenetic topologies, including trees with a backbone structure based on Fukami et al. [19], Kitahara et al. [22], Kerr [20], and another recent composite tree developed by Budd et al. [23]. Each of those backbone trees was supplemented with information on *Acropora* and *Porites* as detailed above, and taxa not present in any of the trees were included or excluded to examine the effects of their placement. The results of this tree exploration showed that the mosaic pattern of multiple independent evolutionary origins of high BRI was present in all phylogenies examined, and a significant association of BRI with scattering coefficient was found in nearly all trees.

Phylogenetic patterns of character change were analyzed using the phylogenetic analysis software package Mesquite (http://mesquiteproject.org). A character by taxon matrix was assembled that included all species of coral in the study as taxa and two matrices of character values; (i) one including continuous quantitative data on bleaching susceptibility (BRI) and reduced light-scattering coefficients (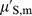) and (ii) another including discrete characters dividing continuous variables into classes of high, medium and low bleaching susceptibility (BRI) and reduced light-scattering coefficients (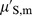) (see clustering analysis section 1.4.2. in Text S1). Evolutionary analysis of character change (such as that shown in Fig. 4) was performed by optimizing the discrete character states of bleaching susceptibility onto the phylogenetic tree, and using the Mirror Tree module of Mesquite to illustrate a pair of characters (BRI and 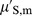) and their associations among coral clades. Quantitative analysis of character correlation was performed using the PDAP (Phylogenetic Diversity Analysis Package) module of Mesquite, which computes the independent contrasts correlation between pairs of variables. Independent contrasts tests account for patterns of phylogenetic relatedness in statistical analysis, allowing us to compute a phylogenetically corrected correlation coefficient and associated significance level for the association of bleaching susceptibility with light-scattering properties.

### Linear regression analysis of potential confounding of growth form

Because of the known relationship between growth form and bleaching susceptibility, where branching forms show generally higher susceptibility to bleaching than massive forms [51], [52], we determined the significance of different pair-wise correlations after accounting for the potential confounding of colony morphology by using robust linear regression analysis (described in detail in Text S1 section 1.5.).

## Supporting Information

Figure S1
**Light-scattering** 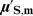 **of 150 coral skeletons (average ± stdev) within the photosynthetically active radiation (∼450 to 670 nm).** Light-scattering varied considerably among the skeletons sampled ranging from 3.02 to 24.39 mm^−1^. This variability could not be explained by measurement uncertainty alone suggesting inherent differences in light transport among coral taxa independently of their geographic distribution.(TIF)

Figure S2
**Absorption (** 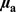 **) and scattering coefficients (** 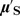 **) of coral skeletons measured using integrating sphere setup.** Solid lines are averages of 22 skeletons and dotted lines are ±1 standard deviation of the mean.(TIF)

Figure S3 **Integrating sphere schematic for the measurement of light-amplification.**
(TIF)

Table S1 **Properties and bleaching response index (BRI) of the 150 skeletons studied.**
(XLS)

Table S2 **List of 1,412 entries used to determine the taxon-specific bleaching response index (BRI) of the 96 taxa studied.**
(XLS)

Table S3 **Source information used to determine taxon-specific bleaching response index (BRI). Data are the compilation of 1,412 entries.**
(XLS)

Table S4 **Coefficients used to convert Gleason 1993 [56] bleaching response dataset into bleaching response index (BRI) used in this study.** (a) Bleaching level (% colony affected) in April 1991 in Gleason 1993, (b) conversion factors - averages of each category for April 1991 bleaching level from Table S4a, (c) bleaching response index (BRI, %) calculated from multiplying % colony affected (Table S4a) by conversion factors (Table S4b) and dividing by sum of categories (Table S4a).
(XLS)

Text S1 **Complete methods.**
(DOC)
