# Supplementary figures and images for: Modulation of Light-Enhancement to Symbiotic Algae by Light-Scattering in Corals and Evolutionary Trends in Bleaching
Source: PLoS One. 2013 Apr 22;8(4):e61492. doi: 10.1371/journal.pone.0061492 (PMC3632607; doi:10.1371/journal.pone.0061492)

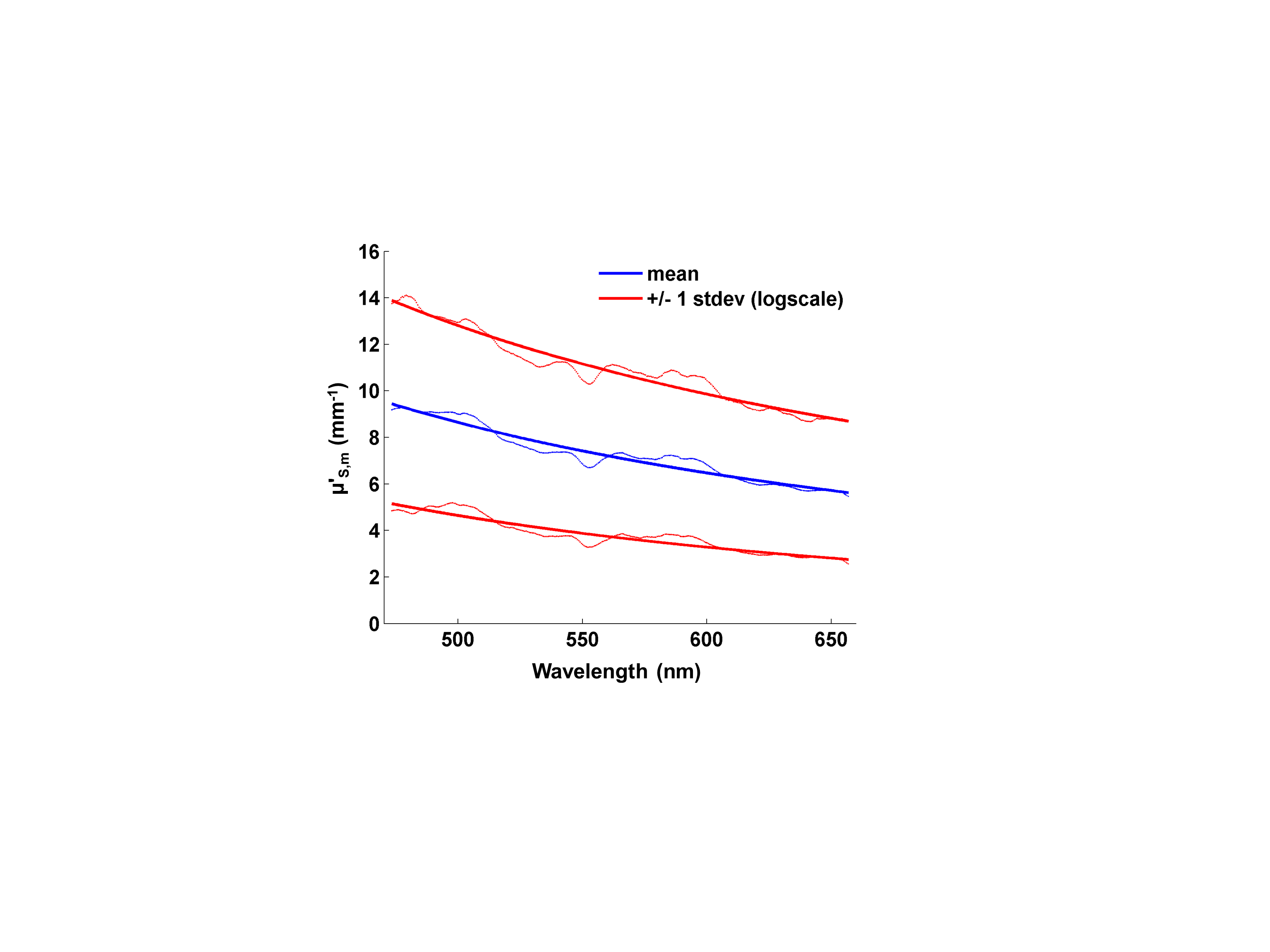

Supplement: Figure S1 — Light-scattering of 150 coral skeletons (average ± stdev) within the photosynthetically active radiation (∼450 to 670 nm). Light-scattering varied considerably among the skeletons sampled ranging from 3.02 to 24.39 mm−1. This variability could not be explained by measurement uncertainty alone suggesting inherent differences in light transport among coral taxa independently of their geographic distribution. (TIF) [file pone.0061492.s001.tif]

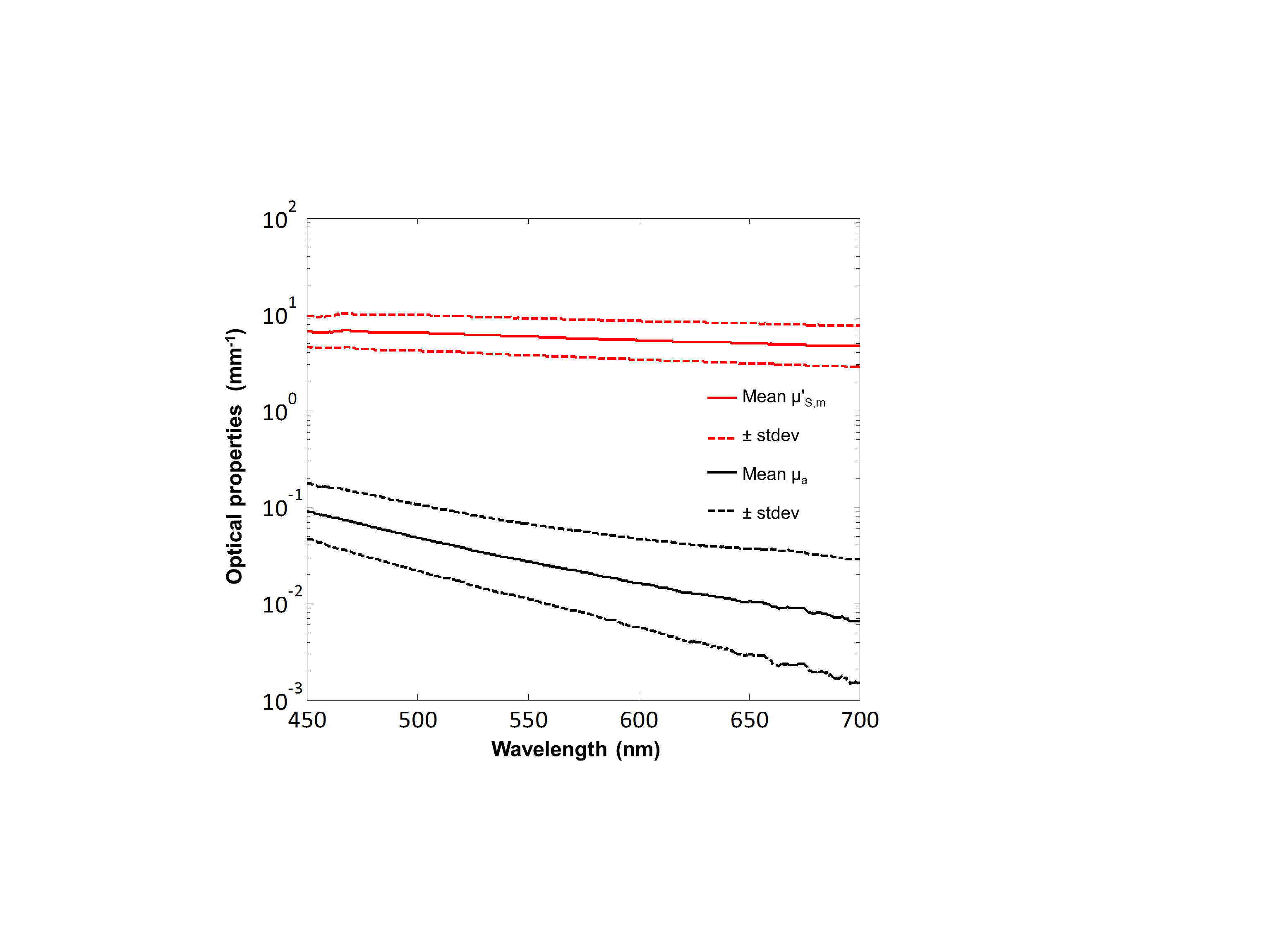

Supplement: Figure S2 — Absorption ( ) and scattering coefficients ( ) of coral skeletons measured using integrating sphere setup. Solid lines are averages of 22 skeletons and dotted lines are ±1 standard deviation of the mean. (TIF) [file pone.0061492.s002.tif]

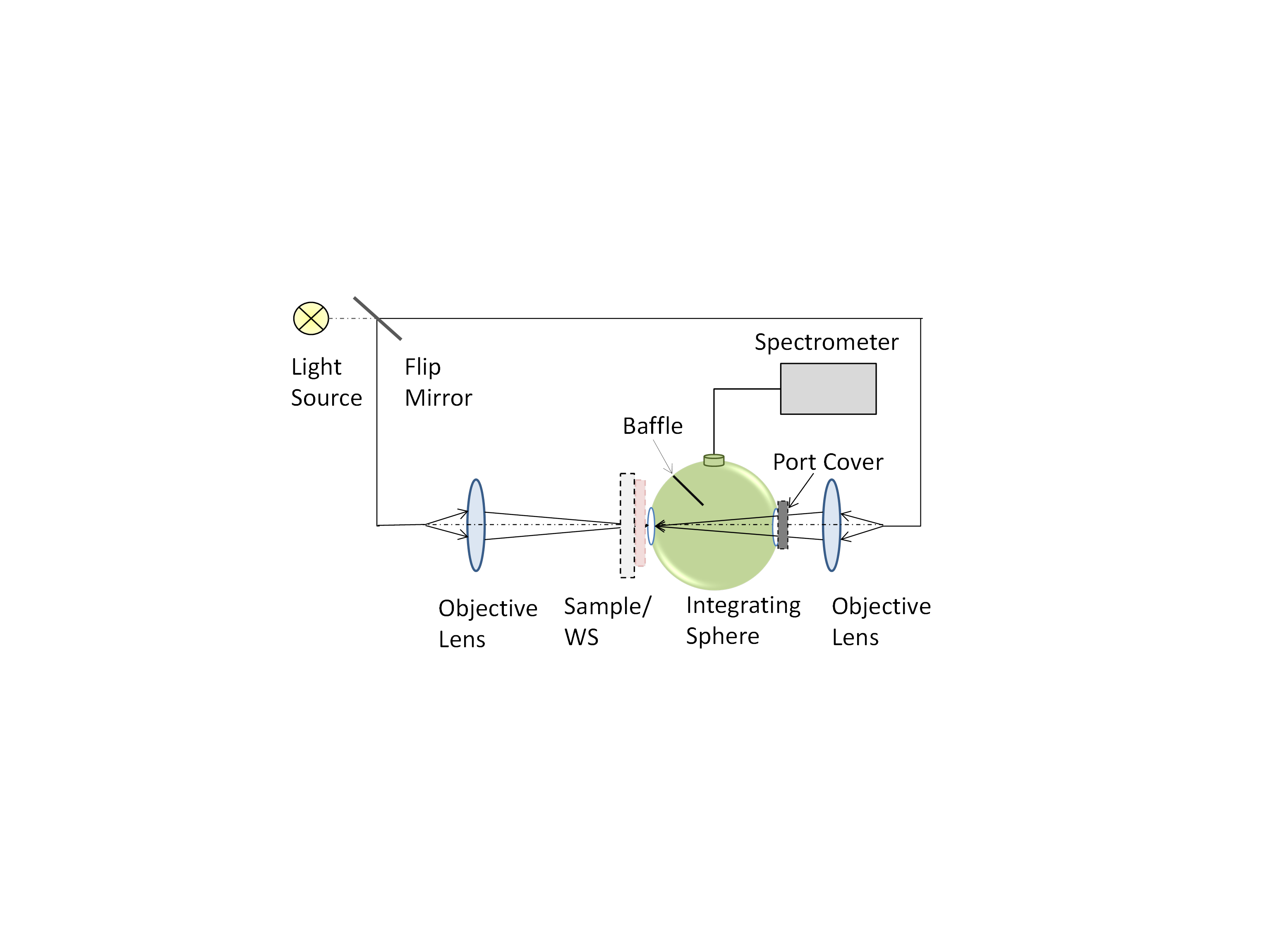

Supplement: Figure S3 — Integrating sphere schematic for the measurement of light-amplification. (TIF) [file pone.0061492.s003.tif]
