# Supplementary material for: Modulation of Light-Enhancement to Symbiotic Algae by Light-Scattering in Corals and Evolutionary Trends in Bleaching
Source: PLoS One. 2013 Apr 22;8(4):e61492. doi: 10.1371/journal.pone.0061492 (PMC3632607; doi:10.1371/journal.pone.0061492)
Supplement: Text S1 — Complete methods. (DOC) [file pone.0061492.s008.doc]

**Light-scattering properties of corals: evolutionary trends in bleaching and enhancement of light absorption by symbiotic algae**

Luisa A. Marcelino^1,2 *^, Mark W. Westneat^2^, Valentina Stoyneva^3^, Jillian Henss^1,2^, Jeremy D. Rogers^3^, Andrew Radosevich^3^, Vladimir Turzhitsky^3^, Margaret Siple^1,2^, Andrew Fang^1^, Timothy D. Swain ^1,2^, Jennifer Fung^1^, Vadim Backman^3^

^1^ Department of Civil and Environmental Engineering, Northwestern University, Evanston, Illinois, United States of America

^2^ Department of Zoology, Field Museum of Natural History, Chicago, Illinois, United States of America

^3^ Department of Biomedical Engineering, Northwestern University, Evanston, Illinois, United States of America

* Email: [l-marcelino@northwestern.edu](mailto:l-marcelino@northwestern.edu)

**Supporting Information**

**1. Methods**

Coral skeletons representing 96 Atlantic and Indo-Pacific taxa (Table S1) were obtained from the Field Museum (Division of Invertebrates; n= 91) and the US National Museum of Natural History (Department of Invertebrate Zoology; n=59). Many corals obtained from the Field Museum collections are original specimens from the Chicago World's Fair: Columbian Exposition of 1893 (highlighting the current value of preserving historical specimens). These specimens were used in the following analyses of optical properties.

*1.1. Low Coherence Enhanced Backscattering (LEBS) spectroscopy*

The enhanced backscattering (EBS) phenomenon originates from the constructive interference of photons traveling time-reversed paths and is observed as an angular intensity cone centered in the backscattering direction [18,53]. LEBS facilitates the measurement of microscopic-scattering through implementation of a broadband partial spatial coherence illumination which selectively isolates the scattering spectrum from the short photon path lengths which remain within the spatial coherence length of the incident light *Lsc*.

The LEBS instrument has been described in detail in other publications [13,18,42]. Briefly, linearly polarized collimated broadband illumination is delivered onto the surface of a given coral skeleton sample at 15º angle of incidence to prevent the collection of the specular reflection. The light backscattered by the sample is collected using a lens, a polarizer, and an imaging spectrograph coupled with a CCD camera. The CCD records a matrix of light-scattering intensities, I_LEBS_(θ,λ), as a function of wavelength λ (from 450 to 700 nm with 0.50 nm resolution) and backscattering angle θ (from –5^o^ to 5^o^ with the resolution of 0.024^o^). The spatial coherence length of illumination, Lsc, can be varied by means of a set of apertures mounted on a rotating wheel in the Fourier plane of illumination arm [18]. In this work, we used a fixed Lsc of ~57 microns at 600 nm illumination [42].

*1.2. Measurement uncertainty*

*1.2.1.* *Reduced scattering coefficient* $\mu'_{\text{S, m}}$

Light-scattering varied considerably among the 150 coral skeletons sampled with $\mu'_{\text{S, m}}$ ranging from 3.02–24.39 mm^-1^ (Table S1). This variability could not be explained by measurement uncertainty due to finite spatial sampling of coral colonies or a limited number of skeletal specimens per species and per geographic location; the measurement uncertainty explained only 38% of the data variance. The variability in $\mu'_{\text{S, m}}$ suggests inherent differences in light transport among coral taxa.

In order to estimate the variance of $\mu'_{\text{S, m}}$ due to the uncertainty of measurements, we used error propagation analysis in the following hierarchy: 1) to determine a $\mu'_{\text{S, m}}$ value for a given coral species, a number of colonies (Nc) per species were measured; 2) to determine $\mu'_{\text{S, m}}$for a given colony, a number of pieces (Np) from the colony were measured; 3) to determine $\mu'_{\text{S, m}}$for a particular piece, a number of sites (Ns) within 1 cm diameter from the same piece were measured. We determined the average standard deviations for each of the hierarchy of measurements: the inter-site standard deviation (data set included all 150 skeletons, 20 sites per piece), the inter-piece standard deviation (a representative subset consisting of skeletons from 7 coral colonies, 4 pieces per colony), and inter-colony standard deviation (a representative subset consisting of colonies from 7 species, 4 to 8 colonies per species). For the data set of 150 coral skeletons corresponding to 96 taxa the average values of colonies, pieces and sites were Nc = 1.56, Np = 1, and Ns = 20, respectively.

Each individual measurement of $\mu'_{\text{S, m}}$was highly accurate: the uncertainty of measuring $\mu'_{\text{S, m}}$for a particular location within a colony was low: 3.3% of the mean $\mu'_{\text{S, m}}$for the piece. This could explain only 0.6% of the total variance in $\mu'_{\text{S, m}}$among the coral taxa. The uncertainty of assigning $\mu'_{\text{S, m}}$to a given colony based on a finite number of pieces measured (Np) was also low: 13.2% of the mean $\mu'_{\text{S, m}}$for the colony, which could explain only 9.3% of the total variance in $\mu'_{\text{S, m}}$among the coral taxa. The uncertainty of assigning $\mu'_{\text{S, m}}$to a species based on the measurement of a limited number of colonies per species (Nc) was 24.0% of the mean $\mu'_{\text{S, m}}$for the species, which corresponds to 28.3% of the total variance. Error propagation analysis given the standard deviations and the values of Nc, Np, and Ns showed that the ratio of the variance due to the overall uncertainty of $\mu'_{\text{S, m}}$ measurements to the variance of the entire data set was 38.2%. The major contributor to the uncertainty was not the error of measurement of individual skeletal sites (3.3% of the mean) but a limited number of coral skeletons representing each taxa (150 skeletons corresponding to 96 taxa). Nevertheless, this analysis shows that the major portion (61.8%) of the variance in the $\mu'_{\text{S, m}}$data for the 96 taxa was due to the inherent differences among the taxa in their values of $\mu'_{\text{S, m}}$.

Furthermore, $\mu'_{\text{S, m}}$ showed no dependence on geographic location (Table S1, Australia n = 12, Central Pacific n = 63, Western Indo-Pacific n = 16, Southeast Asia n = 30, Wider Caribbean n = 25 skeletons, ANOVA, p > 0.5).

*1.2.2. Mass-fractal dimension, D_f_*

We estimated the portion of the variance of D_f_ that is due to measurement uncertainty using the same analysis as described in section 1.2.1. The uncertainty of assigning D_f_ to a given colony piece based on a finite number of sites measured (Ns) was low: 2.4% of the mean D_f_ for the piece, which could explain only 1.7% of the total variance in D_f_ among the coral taxa. The uncertainty of assigning D_f_ to a colony based on a limited number of pieces (Np) was 6.7% of the mean D_f_ for the colony, which could explain 11.4% of the total variance in D_f_ among the taxa. Finally, the uncertainty of assigning D_f_ to a species based on the measurement of a limited number of colonies per species (Nc) was 9.3% of the mean D_f_ for the species which corresponds to 16.5% of the total variance in D_f_ among the coral taxa. The portion of the total variance of D_f_ data over all taxa that could be explained by all causes of uncertainty of measurements of D_f_ was 29.6%, so the major portion (70.4%) of the variance in the D_f_ data for the 96 taxa was due to the inherent differences among the taxa in their values of D_f_.

*1.2.3. Bleaching response index (BRI)*

An approach analogous to the one described in section 1.2.1 was also used to estimate the uncertainty of BRI (see section 1.4.1. for BRI construction) that depends on the uncertainty of bleaching and mortality data from each report and the number of reports for each species. The uncertainty of bleaching and mortality data from a given report was due to the categorization/precision of reported bleaching data. For example, while some studies reported percent bleached cover in increments of 10% others reported it in three categories: pale, bleached, or dead. The error propagation analysis showed that the portion of the total variance of BRI values due to the uncertainty was 22.4%. The uncertainty of assigning a BRI number to a taxon due to the uncertainty/precision of measurements in each individual report was 21.5% of the mean BRI for individual reports which could explain 1.8% of the total BRI variance for all coral taxa. The uncertainty of measurements due to the variability among different reports and a limited number of these reports was 21.6% of the mean BRI for different reports which could explain 20.6% of the total BRI variance for all coral taxa. As we can see, while the precision of reported data in a given report was modest (21.5% of the mean), this did not translate into a significant increase in the uncertainty of BRI (1.8% of the total variance) because of a large number of reports being included in the estimation of a BRI for a given taxon. The major contribution to the overall uncertainty (77.6%) was due to the variability among different reports.

*1.3. Measuring light amplification to the algae using a ‘flat-coral model’*

*1.3.1. Mathematical model of light amplification*

There are two related properties that are used interchangeably to quantify the process of light amplification: light amplification *A* and excess light *E*. Following [13], in this work, light amplification *A* is defined as the ratio of light intensity interacting with symbionts in the presence of a skeleton ($I_{t+substrate}$) to that of without the skeleton ($I_{t}$). Excess light *E* is defined as the difference between light intensities interacting with symbionts with and without a skeleton normalized by the intensity without the skeleton.

$A=\frac{I_{t+substrate}}{I_{t}}$, $E=\frac{I_{t+substrate}-I_{t}}{I_{t}}$ , $A=E+1$.

As shown in [13] and discussed below, in a “flat coral model” (i.e. coral tissue situated on top of a flat, semi-infinite coral skeleton), *A* can be as high as 3. *A=*1 would be achieved in case of a complete absence of the reflection from a substrate (i.e. the skeleton). If after propagating through a layer of tissue, the light that is not absorbed by symbionts were to be reflected back by a perfect mirror, it would pass through the tissue a second time allowing for the second absorption and *A =* 2 would be observed (a similar light amplification mechanism is used in nature in the retinae of some nocturnal animals). Amplification above this level and up to *A* = 3 is only possible due to a skeleton being a diffuse reflector. *A >* 3 can be observed for non-flat morphologies (this bears analogy with multiple reflection-based resonant cavities utilized in laser technology).

The dependence of *A* on the concentration of light absorbing symbionts *ρ* (number surface density) is described by the following equation:

$A = 1 + e^{-\tau}\frac{\left(1\left. - \int_{0}^{2\pi} d\phi\int_{0}^{\pi} p \left(\theta,\left.\phi\right)\right.d\theta\right)e^{\frac{^{-\tau}}{\cos\left(\theta\right)}}\right.}{\left(1 - e^{-\tau}\right)}R = 1 + e^{-\tau}\frac{\left(1-e^{-a\left(\tau\right)\tau}\right)}{\left(1-e^{-\tau}\right)}R$ (1)

where a spatially uniform illumination is assumed, the integration is performed in the spherical coordinates θ and ϕ over all angles, *p*(*θ*,*ϕ*) is the angular distribution of the light interacting with the absorbers normalized such that its total integral equals 1. *τ* = *ρ C_a_* is the optical thickness of the symbiont containing tissue, where *C_a_* is the absorption cross section of a single absorber. *R* is the total reflectance of the skeleton, and

$\alpha\left(\tau\right) = - \frac{1}{\tau} \ln\int_{0}^{2\pi} d\phi\int_{0}^{\pi} p \left(\theta,\phi\right) e^{\frac{-\tau}{\cos\left(\theta\right)}}d\theta$ (2)

quantifies an average elongation of a ray path through tissue after the ray is reflected by the skeleton. Eq. 1 may be used for essentially any skeletal morphology and the effect of the morphology can be quantified by means of function α(τ). One of the reasons α is an important parameter is because in the limit *τ* = *ρ C_a_* << 1, i.e. a bleaching response,

(3)

In the special case of a ‘flat-coral model’, where a coral is approximated as an absorbing, weakly-scattering layer of tissue on top of a semi-infinite light scattering skeleton, the integration in Eq. (1) should be performed over the backscattering directions only (*θ* ≤ π/2) and

(4)

where constant *B* ~ 4.6 [54]. From Eq.4 it can be seen that R increases with the reduced scattering coefficient of the skeleton *µʹ_s_* and decreases with the absorption coefficient *µ_a_*, in agreement with reference [14]. If *µ_a_* = 0, then *R* = 1 for any value of *µʹ_s_* and *A* is a function of τ only and depends on the angular distribution of light emerging from the skeleton after being diffusely reflected back into tissue. For the special case of a skeleton as a Lambertian reflector, α = 2 and for a flat coral model in the limit τ << 1, we arrive to an equation that is in agreement with reference [13]:

$A=1+2R$ (5)

Thus, for a flat coral model *A* can be as high as 3. More complex skeletal geometries, however, allow for a higher α and can lead to *A* as high as 10–20, in agreement with references [9,10].

*1.3.2. Measuring optical properties and light amplification using an integrating sphere technique*

The optical properties of the tissue models were characterized using the integrating sphere (IS) technique similar to [2,14] in combination with the Inverse Adding-Doubling (IAD) method [46–48] based on the diffusion approximation and radiative transport theory. Through the measurement of the normalized transmission and reflection from a thin sample, the IAD program calculates the scattering and absorption coefficients: IAD solves for the unknown optical properties of the sample with measured reflected and transmitted components by guessing dimensionless values related to the desired properties and iteratively solving the radiative transport equation until the solution matches the measured values.

Using the setup shown in Fig. S3, transmission (T) and reflection (R) were measured using a Labsphere integrating sphere (IS-060-SF), light source and spectrometer (Ocean Optics, USB4000) connected by optical fiber to the detector port of the sphere. The incident light is directed by a flip mirror into one of two optical fibers and focused by objective lenses at the sample port of the IS. One of the beams passes through the IS to the sample to measure reflection while the other is used to measure the total transmission with back port covered. Two HeNe lasers (Melles Griot) emitting at 543 nm and 633 nm were used to measure the optical properties of the fluorescent slab to assure no influence of the fluorescent signal. By illuminating only at the wavelength of the measurement, fluorescence that would otherwise be excited by shorter wavelengths is avoided.

All sample measurements (*R*, *T*) were dark-signal subtracted (*R_0_*, *T_dark_*) and normalized by incident intensity (*T_0_*) for transmission measurements, or by the reflectance of a reflectance standard (“white standard”, *ws*):

Reflectance-measurement $=r_{ws}\frac{R-R_{0}}{R_{ws}-R_{0}}$ ,Transmittance-measurement $=\frac{T-T_{dark}}{T_{0}-T_{dark}}$, whereas $R_{ws}$ is the signal measured when the white standard was placed at the sample port of the IS. The reflectance of the reference plate, r_ws_ = 0.99, was provided by the manufacturer. Five sample readings were recorded for each of the parameters with sample orientation randomized.

The excess light (*E*) (i.e. the difference between light intensities interacting with symbionts with and without a skeleton normalized by the intensity without the skeleton) was determined using the same IS setup in the reflection mode. He-Ne laser (543 nm) was used for illumination to target the excitation maximum of the fluorescent microspheres and to allow clear separation of the absorption and emission spectra.

First the tissue-mimicking layer was placed at the sample port of the IS and the intensity of the fluorescent signal, $I_{t}$, recorded. Then a coral skeleton slab on top of the white standard (hereafter, ‘substrate’) was placed under the tissue-mimicking layer and the fluorescence spectrum was measured, $I_{t+substrate}$. The presence of the white standard ensures that the measurements were not confounded by the finite thickness of the skeleton slabs. The excess light *E* was then calculated by taking the relative increase in the fluorescent signal normalized by the signal measured without the presence of skeleton-white standard system averaged over the wavelength range 580–620 nm.

$$E=\left\langle\frac{I_{t+substrate}-I_{t}}{(1+R_{substrate})(I_{t}-I_{dark})}\right\rangle$$

Where $I_{dark}$ is the measured intensity with tissue layer present at the measurement port and the incident light blocked and $R_{substrate}$ is the reflectance of the skeleton-white standard combination measured at the fluorescence emission wavelengths. The factor of (1+$R_{substrate}$) comes from the fact that when the fluorescence from the tissue-coral skeleton slab-white standard model is acquired, in addition to the ‘direct fluorescence’ from the microspheres (i.e. fluorescence emitted by the microspheres in the tissue-mimicking layer and directly measured by the detector), the total collected fluorescence signal has also a diffuse component. This component after being emitted by the fluorescence microspheres propagates to the skeleton and is then reflected by it back to the detector by means of diffuse scattering.

In order to relate the excess light interacting with fluorescent microspheres and the microscopic-scattering properties of a reflective substrate the reduced scattering coefficient of the entire substrate (i.e. coral skeleton on top of a white standard;$\mu'_{\text{S,m}}$ in Fig. 2B) must be determined. Although the substrate $\mu'_{\text{S,m}}$ is related to that of the skeleton slab ($\mu'_{\text{S, skeleton}}$), they are not identical because of the effect of the white standard, which typically has a much greater reduced scattering coefficient, $\mu'_{\text{S, WS}}$. Substrate $\mu'_{\text{S,m}}$was estimated as follows:

,

where $R_{skeleton}$and $T_{skeleton}$are the reflectance and the transmittance of the coral skeleton slice alone (without the white standard underneath) measured at the absorption wavelengths.

Figure 2B shows the rate of excess light increase Δ*E* as a function of the LEBS reduced scattering coefficient of the substrate $\mu'_{\text{S,m}}$: linear regression $\Delta E=0.50-0.0043\mu'_{\text{S,m}}[mm^{-1}]$, *R*^2^ = 0.66, p-value = 0.0008. Of note is that the use of the LEBS reduced scattering coefficient of the skeleton samples without being corrected for the effect of the underlying white reflectance standard ($\mu'_{\text{S,skeleton}}$) gives a similar trend: linear regression $\Delta E=0.45-0.0033\mu'_{\text{S,skeleton}}[mm^{-1}]$, *R*^2^ = 0.68, p-value = 0.0005.

*1.3.3. Absorption coefficient of coral skeletons*

The potential confounding effect of the skeletal absorption on the excess light relation with the scattering properties was investigated. The optical properties of a subset of 22 skeletons randomly chosen from the collection of 150 skeletons were measured with the integrating sphere technique and the scattering and absorption coefficients of the skeleton slices were calculated with IAD algorithm as described above. White light (Xenon Lamp, Thorlabs) was used for illumination to assess the optical properties across the visible spectrum (450 to 700 nm). The absorption coefficient $\mu_{a}$ was over 100 times smaller than the reduced scattering coefficient as can be seen in Fig S2. This means that over a pathlength comparable to transport mean free path ($l'_{\text{S, m}}$), only 0.5% of the light would be absorbed by the skeleton. Furthermore, there was no significant correlation between $\Delta_{E}$ and $\mu_{a}$ (linear regression p > 0.5).

*1.4. Bleaching response index (BRI)*

*1.4.1. Construction of BRI*

Using taxon-specific data from mass bleaching events throughout the tropics from 1982–2005 that were previously published in peer-review, gray literature, and electronic databases (Table S3), we compiled 1,412 unique records of bleaching severity and related mortality (Table S2). All collected data were converted to a bleaching response index (BRI) which was defined as the taxon-specific average percent coral cover affected during mass bleaching events, i.e. coral colonies found bleached and/or dead. We reasoned that this index provides a measure of susceptibility to bleaching without being confounded by (i) differential susceptibility to bleaching-induced mortality among corals, which can be modulated by factors not related to bleaching per se, (e.g. access to nutrition [55]) nor (ii) time of observation relative to the onset of an episode which could result in a higher frequency of bleaching if observed earlier or higher frequency of death if observed later.

While some studies reported their data on a linear severity score, others used a perceived severity of bleaching and death by relating weighing coefficients to a particular level of bleaching response. In the latter reports, the rate of increase of the weighing coefficients as a function of % of bleached cover decreases with severity; i.e. the contribution from the least bleached corals is increased relatively to the most bleached corals (Table S3). In order to include these reports, we developed an empirical relationship between the score of perceived severity of bleaching response and the percentage of affected coral cover. Among nearly all reports, this relationship is proportional to the square root of the fraction of the affected corals. For surveys that reported the number of bleached and/or dead colonies per categories of bleached and/or dead cover, we first estimated the expectation of the affected coral cover for each category and then the average BRI was calculated according to equation (6),

$BRI=\sum_{i} p_{i}C_{i}+p_{D}=\frac{\sum_{i} N_{i}C_{i}+N_{D}}{\sum_{i} N_{i}+N_{D}}$ (6)

where $p_{i}=\frac{N_{i}}{\sum_{i} N_{i}+N_{D}}$is the probability (or percentage of colonies) in affected category i, $C_{i}$= % of bleached cover in category i, $N_{i}$= number of colonies in category i and $N_{D}$= number of dead colonies ($C_{D}=1$). As an example let’s use the bleaching and mortality data reported by Gleason 1993 [56], compiled in Table S4a. Gleason’s initial survey in April 1991 right after the bleaching episode reported extensive bleaching with not yet noticeable mortality for 8 taxa. The expectation of the affected coral cover for each category was calculated (Table S4b), and these $C_{i}$ were multiplied by the reported values (% of affected colonies in [56]) to calculate the average BRI (Table S4c). A later survey in August 1991 accessed the recovery for the same 8 taxa and reported significant bleaching and mortality. BRI values for the second time point were calculated separately and the average BRI for April and August 1991 was included in the final dataset (entries ‘Gleason 1993’, Table S2).

For surveys that did not explicitly provide the fraction of either bleached or dead corals, these were inferred based on the available data. Studies reporting only taxa-specific bleaching without mortality information were not included. All BRI values from the individual surveys (Table S2) were then averaged to yield taxon-specific BRI for the 96 taxa in this study (Table S1). Skeletons belonging to a given species were assigned a particular BRI score based on the species-level information if there were at least 3 independent reports (average 10.35 records/species, 52 species with species-level information); otherwise a score with genus-level information was assigned (average 27.93 records/genus, 44 taxa with genus-level information).

A limitation of our BRI is that it is an accurate measure of the percent of bleached coral cover only if two conditions are satisfied. Firstly, non-bleaching related deaths (e.g. infectious diseases) are neglected. Secondly, probability of bleached corals to recover between the bleaching episode and the time of survey is neglected. Given that most surveys are performed soon after a bleaching episode, both approximations are reasonable and the probabilities of coral recovery and non-bleaching mortality are expected to be small compared to the probability that a coral will either not bleach or bleach and/or die due to bleaching [57,58]. Furthermore, the time of the survey in relation to the bleaching event determines the type of mortality that each study measures: while surveys of mortality taken within weeks of bleaching are more likely to identify “bleaching-induced” mortality (wherein corals show tissue damage and die soon after bleaching), mortality surveys that take place weeks to months after bleaching are more likely to capture “bleaching-related” mortality, which is due to post-bleaching predation and increased susceptibility to disease and bioerosion [59,60]. However, since the distinction between both types of mortality is sometimes difficult we included both types of surveys in our analysis which, although increases the uncertainty of the taxon-specific BRI, was determined to explain only 22.4% of the BRI variance (see SI section 1.2.3).

*1.4.2. Clustering Analysis*

Taxa were further assigned to a number of severity categories that were defined based on clustering analysis for BRI (Mathematica, k-clustering algorithm). Firstly, we determined the number of clusters that should be used by performing a receiver observer characteristic (ROC)-based analysis. The goal of the ROC analysis was to determine the number of clusters that can be identified given an inherent uncertainty in the determination of BRI values for any given species (due for example to a limited number of reports, see section 1.2.3.). A more conventional algorithm that is implemented as part of the k-clustering method and is used to determine the number of clusters is less efficient because it does not take into account uncertainty of the data that is critical if the clusters are to be used for evaluation of accuracy of a classifier (e.g. light scattering properties such as $\mu'_{\text{S, m}}$). We perturbed the BRI values from the original data set using a random number generator to model 100 realizations of BRI values that obeyed the same probability distribution function as the original data set with uncertainty consistent with those for the original data set (uncertainty ~ 22.4% of the data variance, see section 1.2.3.). We then calculated areas under the ROC curves (AUC) for a binary classification (a high BRI group versus a low BRI group) for each of the 100 synthetic data sets by comparing their values with those of the original data set that we considered to be the ‘true’ BRI values. The values of AUC were then plotted as a function of the classifier threshold (i.e. high BRI versus a low BRI). The average of all 100 AUC functions resulted in an average AUC. We found that the majority of the AUC functions had an inverted top-head shape with the average AUC function having a U-shape suggesting that three clusters can be identified given the level of variability and the probability distribution of the data.

Secondly, k-clustering with a squared Euclidean distance was performed to identify three clusters in the BRI data set. The clusters were as follows: low severity (31 taxa, BRI = 18.42 ± 0.82%, mean ± SE), medium severity (48 taxa, BRI = 36.35 ± 0.53%), and high severity (17 taxa, BRI = 57.27 ±1.5%). The clustering algorithm resulted in the medium bin comprising species with BRI within exactly ± 2/3 standard deviations from the mean of the entire data set.

Finally, for the phylogenetic mapping of $\mu'_{\text{S, m}}$ onto the topology tree (see methods), we grouped the 96 taxa according to their $\mu'_{\text{S, m}}$ values into three clusters following the same relative thresholds as in case of the BRI-based clusters with the medium-$\mu'_{\text{S, m}}$ clusters including coral species with $\mu'_{\text{S, m}}$ values within +/-2/3 standard deviations from the mean of $\mu'_{\text{S, m}}$ for the entire data set.

*1.5. Linear regression analysis of potential confounding of growth form*

We show in the results that the optical ($\mu'_{\text{S, m}}$) and structural (D_f_) characteristics of skeletons are correlated with both growth forms and bleaching response. However, because of the known relationship between growth form and bleaching susceptibility, where branching forms show generally higher susceptibility to bleaching than massive forms [51,52] we determined the significance of different pair-wise correlations after accounting for the potential confounding of colony morphology by using robust linear regression analysis.

To remove the potential confounding of colony morphology, each species was assigned an index based on its morphology (1 through 5 for massive, laminar, thick-, medium- and thin-branching morphologies, respectively). Linear regression between a parameter under consideration ($\mu'_{\text{S, m}}$, D_f_, BRI) and the morphology index was performed with the output being regression coefficients, which were then used to subtract out the effect of morphology from the regression parameter. The resulting morphology-corrected parameters were used in a follow-up regression analysis to establish the pair-wise correlations. The analysis showed that after accounting for coral morphology, pair-wise correlation remained significant: $\mu'_{\text{S, m}}$and BRI, p < 0.01 (inverse relationship described in Fig. 5); D_f_ and BRI, p <0.01.

To further confirm the lack of confounding, a secondary analysis was based on multiple linear regression analysis where colony morphology was added to the pair-wise correlates with BRI being the dependent variable and$\mu'_{\text{S, m}}$, D_f_ and growth form the independent predictors. Regression for$\mu'_{\text{S, m}}$, D_f_ remained significant (p < 0.05 and p < 0.01, respectively).

*1.6. Analysis of correlation between excess light E and BRI*

Excess light *E* and $\Delta_{E}$, which is the rate of increase of E with a decrease in the concentration of symbionts (ρ), describe physiological different processes and are determined by different sets of the optical properties of coral skeletons. This is one of the key concepts of the paper. *E* quantifies the level of excess light interacting with symbionts due to the presence of a coral skeleton for a given concentration of the symbionts. The rate of excess light quantifies how fast *E* rises when symbiont concentration is decreased thus exposing the remaining symbionts to even higher light intensities. Thus, $\Delta_{E}$ quantifies the strength of a positive feedback-loop between the reduction in the symbionts’ concentration and the light amplification.

As discussed in section 1.3.1., for a given concentration of symbionts, *E* depends on the total diffuse reflectance of a coral skeleton *R* and in the limit of small symbiont concentration, *E =* 2*R.* In turn, *R* depends on the ratio of the reduced scattering $\mu'_{\text{S}}$ and absorption $\mu_{a}$ coefficients of the skeleton. For comparison, $\Delta_{E}$ primarily depends on the reduced scattering coefficient, because the typical skeletal values of $\mu_{a}$ are too small (see section 1.3.3.) to significantly affect light absorption over the transport mean free path lengths.

Our experimental data indicates that $\Delta_{E}$ but not *E* correlates with bleaching susceptibility.  Although reflectance *R* and therefore *E* did correlate positively with BRI, the correlation was weak (correlation coefficient = 0.09) and not statistically significant (p = 0.26). Furthermore, the difference in *R* among the 3 BRI clusters (low, medium, and high) was not significant (p > 0.6).

**2. References**

1. Muscatine L (1990) The role of symbiotic algae in carbon and energy flux in reef corals. In: Dubinsky Z ed. Ecosystems of the World 25, Coral Reefs. Amsterdam: Elsevier. pp 75–87.

2. Stambler N, Dubinsky Z (2005) Corals as light collectors: an integrating sphere approach. Coral Reefs 24: 1–9.

3. Dubinsky Z, Falkowski PG, Porter JW, Muscatine L (1984) Absorption and utilization of radiant energy by light- and shade-adapted colonies of the hermatypic coral *Stylophora* *pistillata*. Proceedings of the Royal Society of London Series B-Biological Sciences 222: 203–214.

4. Muko S, Kawasaki K, Sakai K, Takasu F, Shigesada N (2000) Morphological plasticity in the coral *Porites sillimaniani* and its adaptive significance. Bulletin of Marine Science 66: 225–239.

5. Kaniewska P, Anthony KRN, Hoegh-Guldberg O (2008) Variation in colony geometry modulates internal light levels in branching corals, *Acropora humilis* and *Stylophora pistillata*. Marine Biology 155: 649–660.

6. Salih A, Larkum A, Cox G, Kühl M, Hoegh-Guldberg O (2000) Fluorescent pigments in corals are photoprotective. Nature 408: 850–853.

7. Schlichter D, Fricke HW, Weber W (1986) Light harvesting by wavelength transformation in a symbiotic coral of the Red Sea twilight zone. Marine Biology 91: 403–407.

8. Kühl M, Cohen Y, Dalsgaard T, Jørgensen BB, Revsbech NP (1995) Microenvironment and photosynthesis of zooxanthellae in scleractinian corals studied with microsensors for O_2_, Ph and light. Marine Ecology Progress Series 117: 159–172.

9. Enríquez S, Méndez ER, Iglesias-Prieto R (2005) Multiple scattering on coral skeletons enhances light absorption by symbiotic algae. Limnology and Oceanography 50: 1025–1032.

10. Enríquez S, Méndez E, Hoegh-Guldberg O, Iglesias-Prieto R. (2008) Morphological dependence of the variation in the light amplification capacity of coral skeleton. ICRS, 5-18, Fort Lauderdale, FL.

11. Lesser MP, Farrell JH (2004) Exposure to solar radiation increases damage to both host tissues and algal symbionts of corals during thermal stress. Coral reefs 23: 367–377.

12. Bhagooli R, Hidaka M (2004) Photoinhibition, bleaching susceptibility and mortality in two scleractinian corals, *Platygyra ryukyuensis* and *Stylophora pistillata*, in response to thermal and light stresses. Comparative Biochemistry and Physiology Part A: Molecular & Integrative Physiology 137: 547–555.

13. Roy H, Turzhitsky V, Kim Y, Goldberg M, Watson P, et al. (2009) Association between rectal optical signatures and colonic neoplasia: Potential applications for screening. Cancer Research 69: 4476–4483.

14. Terán E, Méndez ER, Enríquez S, Iglesias-Prieto R (2010) Multiple light scattering and absorption in reef-building corals. Applied Optics 49: 5032–5042.

15. Rogers JD, Çapoğlu IR, Backman V (2009) Nonscalar elastic light scattering from continuous random media in the Born approximation. Optics Letters 34: 1891–1893.

16. Stolarski J (2003) Three-dimensional micro- and nanostructural characteristics of the scleractinian coral skeleton: a biocalcification proxy. Acta Palaeontologica Polonica 48: 497–530.

17. Cuif JP, Dauphin Y (2005) The Environment Recording Unit in coral skeletons - a synthesis of structural and chemical evidences for a biochemically driven, stepping-growth process in fibres. Biogeosciences 2: 61–73.

18. Turzhitsky V, Rogers JD, Mutyal NN, Roy HK, Backman V (2010) Characterization of Light Transport in Scattering Media at Subdiffusion Length Scales with Low-Coherence Enhanced Backscattering. IEEE Journal of Selected Topics in Quantum Electronics 16: 619–626.

19. Fukami H, Chen CA, Budd AF, Collins A, Wallace CC, et al. (2008) Mitochondrial and nuclear genes suggest that stony corals are monophyletic but most families of stony corals are not (Order Scleractinia, Class Anthozoa, Phylum Cnidaria). PLoS ONE 3: e3222.

20. Kerr AM (2005) Molecular and morphological supertree of stony corals (Anthozoa : Scleractinia) using matrix representation parsimony. Biological Reviews 80: 543–558.

21. Forsman ZH, Barshis DJ, Hunter CL, Toonen RJ (2009) Shape-shifting corals: molecular markers show morphology is evolutionarily plastic in *Porites*. BMC Evolutionary Biology 9: 45.

22. Kitahara MV, Cairns SD, Stolarski J, Blair D, Miller DJ (2010) A Comprehensive Phylogenetic Analysis of the Scleractinia (Cnidaria, Anthozoa) based on Mitochondrial CO1 Sequence Data. PLoS ONE 5: e11490.

23. Budd AF, Romano SL, Smith ND, Barbeitos MS (2010) Rethinking the Phylogeny of Scleractinian Corals: A Review of Morphological and Molecular Data. Integrative and Comparative Biology 50: 411–427.

24. Basillais E (1997) Coral surfaces and fractal dimensions: a new method. Comptes Rendus de l'Académie des Sciences - Serie III - Sciences de la Vie 320: 653–657.

25. Kaandorp JA, Sloot PMA, Merks RMH, Bak RPM, Vermeij MJA, et al. (2005) Morphogenesis of the branching reef coral *Madracis mirabilis*. Proceedings of the Royal Society B-Biological Sciences 272: 127–133.

26. Weiner S, Sagi I, Addadi L (2005) Choosing the crystallization path less traveled. Science 309: 1027–1028.

27. Martin-Garin B, Lathuiliére B, Verrecchia EP, Geister J (2007) Use of fractal dimensions to quantify coral shape. Coral Reefs 26: 541–550.

28. Pipich V, Balz M, Wolf SE, Tremel W, Schwahn D (2008) Nucleation and growth of CaCO_3_ mediated by the egg-white protein ovalbumin: a time-resolved in situ study using small-angle neutron scattering. Journal of the American Chemical Society 130: 6879–6892.

29. Alberich-Bayarri A, Marti-Bonmati L, Pérez MA, Sanz-Requena R, Lerma-Garrido JJ, et al. (2010) Assessment of 2D and 3D fractal dimension measurements of trabecular bone from high-spatial resolution magnetic resonance images at 3 T. Medical Physics 37: 4930–4937.

30. Gutierrez JL, Jones CG, Strayer DL, Iribarne OO (2003) Mollusks as ecosystem engineers: the role of shell production in aquatic habitats. Oikos 101: 79–90.

31. Abramovitch-Gottlib L, Dahan D, Golan Y, Vago R (2005) Effect of light regimes on the microstructure of the reef-building coral *Fungia simplex*. Materials Science & Engineering C-Biomimetic and Supramolecular Systems 25: 81–85.

32. Lough JM, Barnes DJ (2000) Environmental controls on growth of the massive coral *Porites*. Journal of Experimental Marine Biology and Ecology 245: 225–243.

33. Carricart-Ganivet JP (2004) Sea surface temperature and the growth of the West Atlantic reef-building coral *Montastraea annularis*. Journal of Experimental Marine Biology and Ecology 302: 249–260.

34. Crabbe MJC (2009) Scleractinian coral population size structures and growth rates indicate coral resilience on the fringing reefs of North Jamaica. Marine Environmental Research 67: 189–198.

35. Nothdurft LD, Webb GE (2007) Microstructure of common reef-building coral genera *Acropora*, *Pocillopora*, *Goniastrea* and *Porites*: constraints on spatial resolution in geochemical sampling. Facies 53: 1–26.

36. Mieog JC, Olsen JL, Berkelmans R, Bleuler-Martinez SA, Willis BL, et al. (2009) The roles and interactions of symbiont, host and environment in defining coral fitness. PLoS ONE4: e6364.

37. Baker AC (2003) Flexibility and specificity in coral-algal symbiosis: Diversity, ecology, and biogeography of Symbiodinium. Annual Review of Ecology, Evolution, and Systematics 34: 661–689.

38. DeSalvo MK, Sunagawa S, Fisher PL, Voolstra CR, Iglesias-Prieto R, et al. (2010) Coral host transcriptomic states are correlated with *Symbiodinium* genotypes. Molecular Ecology 19: 1174–1186.

39. Abrego D, Ulstrup KE, Willis BL, van Oppen MJH (2008) Species-specific interactions between algal endosymbionts and coral hosts define their bleaching response to heat and light stress. Proceedings of the Royal Society B-Biological Sciences 275: 2273–2282.

40. Fitt WK, Gates RD, Hoegh-Guldburg O, Bythell JC, Jatkar A, et al. (2009) Response of two species of Indo-Pacific corals, *Porites cylindrica* and *Stylophora pistillata*, to short-term thermal stress: The host does matter in determining the tolerance of corals to bleaching. Journal of Experimental Marine Biology and Ecology 373: 102–110.

41. Spalding MD, Fox HE, Allen GR, Davidson N, Fernaña ZA , et al. (2007) Marine ecoregions of the world: a bioregionalization of coastal and shelf areas. Bioscience 57: 573–583.

42. Kim YL, Liu Y, Turzhitsky VM, Roy HK, Wali RK, et al. (2004) Coherent backscattering spectroscopy. Optics Letters 29: 1906–1908.

43. Philipson B (1969) Distribution of protein within normal rat lens. Investigative Ophthalmology 8: 258–270.

44. Pogue BW, Patterson MS (2006) Review of tissue simulating phantoms for optical spectroscopy, imaging and dosimetry. Journal of Biomedical Optics 11: 041102.

45. Amoozegar C, Giacomelli MG, Keener JD, Chalut KJ, Wax A (2009) Experimental verification of T-matrix-based inverse light scattering analysis for assessing structure of spheroids as models of cell nuclei. Applied Optics 48: D20–D25.

46. Prahl SA, van Gemert MJC, Welch AJ (1993) Determining the optical properties of turbid media by using the adding-doubling method. Applied Optics 32: 559–568.

47. Prahl S (2012) Inverse Adding-Doubling. Available: <http://omlc.ogi.edu/software/iad/index.html>. Accessed 2 March 2013.

48. Pickering JW, Prahl SA, van Wieringen N, Beek JF, Sterenborg HJCM, et al. (1993) Double-integrating-sphere system for measuring the optical properties of tissue. Applied Optics 32: 399–410.

49. Veron JEN (2000) Corals of the World. Vol. 1–3.Townsville, Australia: Australian Institute of Marine Science. 1382 p.

50. Wallace CC (1999) Staghorn corals of the world: a revision of the genus *Acropora* (Scleractinia: Astrocoeniina: Acroporidae) worldwide, with emphasis on morphology, phylogeny and biogeography. Melbourne, Australia: CSIRO Publishing. 421 p.

51. Loya Y, Sakai K, Yamazato K, Nakano Y, Sambali H, et al. (2001) Coral bleaching: the winners and the losers. Ecology Letters 4: 122–131.

52. Marshall PA, Baird AH (2000) Bleaching of corals on the Great Barrier Reef: differential susceptibilities among taxa. Coral Reefs 19: 155–163.

53. Akkermans E, Wolf PE, Maynard R (1986) Coherent backscattering of light by

disordered media: analysis of the peak line shape. Physical Review Letters

56: 1471–1474.

54. Madsen S, Wilson B, Patterson M, Park Y, Jacques S, et al. (1992) Experimental tests of a simple diffusion model for the estimation of scattering and absorption coefficients of turbid media from time-resolved diffuse reflectance measurements. Applied Optics 31: 3509–3517.

55. Grottoli AG, Rodrigues LJ, Palardy JE (2006) Heterotrophic plasticity and resilience

in bleached corals. Nature 440: 1186–1189.

56. Gleason MG (1993) Effects of disturbance on coral communities: bleaching in

Moorea, French Polynesia. Coral Reefs 12: 193–201.

57. Obura DO (2001) Can differential bleaching and mortality among coral species offer

useful indicators for assessment and management of reefs under stress? Bulletin

of Marine Science 69: 421–442.

58. McClanahan TR, Baird AH, Marshall PA, Toscano MA (2004) Comparing bleaching

and mortality responses of hard corals between southern Kenya and the Great

Barrier Reef, Australia. Marine Pollution Bulletin 48: 327–335.

59. Obura DO (2005) Resilience and climate change: lessons from coral reefs and

bleaching in the Western Indian Ocean. Estuarine, Coastal and Shelf Science 63:

353–372.

60. Jones RJ (2008) Coral bleaching, bleaching-induced mortality, and the adaptive

significance of the bleaching response. Marine Biology 154: 65–80.
